# Genome-wide analysis to identify a novel microRNA signature that predicts survival in patients with stomach adenocarcinoma

**DOI:** 10.7150/jca.33250

**Published:** 2019-10-17

**Authors:** Shan-Shan Luo, Xi-Wen Liao, Xiao-Dong Zhu

**Affiliations:** 1 Department of Gastrointestinal Surgery, Affiliated Tumor Hospital of Guangxi Medical University, Nanning, 530021, Guangxi Zhuang Autonomous Region, People's Republic of China.; 2 Department of Hepatobiliary Surgery, The First Affiliated Hospital of Guangxi Medical University, Nanning, 530021, Guangxi Zhuang Autonomous Region, People's Republic of China.; 3 Department of Radiation Oncology, Affiliated Tumor Hospital of Guangxi Medical University, Cancer Institute of Guangxi Zhuang Autonomous Region, Nanning, 530021, Guangxi Zhuang Autonomous Region, People's Republic of China.

**Keywords:** genome-wide, miRNA, TCGA, stomach adenocarcinoma, prognosis.

## Abstract

**Objective**: Using genome-wide screening, this study was aimed at identifying prognostic microRNA (miRNA) in those patients suffering from stomach adenocarcinoma (STAD).

**Methods**: A genome-wide miRNA sequencing dataset and relevant STAD clinical information was obtained via The Cancer Genome Atlas (TCGA). Prognostic miRNA selection was carried out through a whole genome multivariate Cox regression model in order to establish a prognostic STAD signature.

**Results**: Eleven miRNAs (hsa-mir-509-2, hsa-mir-3917, hsa-mir-495, hsa-mir-653, hsa-mir-3605, hsa-mir-2115, hsa-mir-1292, hsa-mir-137, hsa-mir-6511b-1, hsa-mir-145, and hsa-mir-138-2) were recognized as prognostic and used for the construction of a STAD prognostic signature. This signature exhibited good performance in predicting prognosis (adjusted *P*<0.0001, adjusted hazard ratio= 3.047, and 95% confidence interval=2.148-4.323). The time-dependent receiver operating characteristic examination exhibited area under curve values of 0.711, 0.697, 0.716, 0.733, 0.805, and 0.805, for 1-, 2-, 3-, 4-, 5-, and 10-year overall survival (OS) estimation, respectively. Comprehensive survival analysis suggests that the 11-miRNA prognostic signature acts as an independent feature of STAD prognosis and exhibits superior performance in OS prediction when compared to traditional clinical parameters. Furthermore, fourteen miRNA target genes were linked to STAD OS. These included *SERPINE1, MLEC, ANGPT2, C5orf38, FZD7, MARCKS, PDGFD, DUSP6, IRS1, PSAT1, TENM3, TMEM127, BLMH,* and *TIRAP*. Functional and gene set enrichment analysis suggested that target genes and the 11-miRNA prognostic signature were both participate in various biological processes and pathways, including the growth factor beta, Wnt, and Notch signaling pathways.

**Conclusions**: By means of a genome-wide analysis, an 11-miRNA expression signature that may serve as an underlying prognostic indicator for those patients suffering from STAD has been identified and described here.

## Introduction

With nearly 951,600 new cases and 723,100 deaths occurring due to stomach cancer across the globe in 2012, stomach cancer is considered a critical cause of death. However, the rates of stomach cancer are normally higher in men than in women 1. Moreover, there is a higher rate of stomach or gastric cancer occurrence in Eastern Asia, which includes China, Japan, Korea, and Mongolia 1, than the rest of the world.

The most common histological type of stomach cancer is stomach adenocarcinoma (STAD). The tumorigenesis of stomach cancer is largely driven by means of genetic and environmental factors, with the most well-known etiology occurring through chronic *H. pylori* infection. This bacteria is known to be the main cause of noncardia stomach cancer 2. High levels of salt consumption, together with nitrate and marinated food intake, obesity, and smoking have also been linked with an increased risk of developing stomach cancer 3-5. For genetic factors, The Cancer Genome Atlas (TCGA) has made available the findings of the entire genomic profile of primary STAD, and has also proposed the molecular arrangement that divides stomach cancer into four distinctive subcategories. As per the genomic transformation of multiple genes, this includes phosphatidylinositol-4,5-bisphosphate 3-kinase catalytic subunit alpha (PIK3CA) mutations, extreme DNA hypermethylation, amplification of the Janus kinase 2 (JAK2), and programmed cell death ligand 1 (CD274: also known as PD-L1) and programmed cell death 1 ligand 2 (PDCD1LG2: also referred as PD-L2) 6. Nevertheless, owing to its complexity, the genetic pathogenesis of stomach cancer requires further investigation.

Moreover, to satisfactorily combat stomach cancer, clinical re-sectioning therapy is considered the only curative option 7, 8. However, patients suffering from advanced stage or metastatic stomach cancer have often been unable to undergo reoperation and need to seek alternative treatments. This includes targeted treatments and chemotherapy. Previous studies have reported that microRNA (miRNA) may have theragnostic potential in patients with stomach cancer, which includes chemotherapy and targeted therapies 9-13. Moreover, miRNA also serves as prognostic 14, 15 and diagnostic 16, 17 biomarkers of stomach cancer, and is associated with the progress of stomach cancer 18 and metastasis 19, 20. Therefore, the systematic investigation of the underlining mechanisms and discovery of prognostic miRNA markers for stomach cancer may help in developing targeted treatment strategies and improving clinical outcomes. An open access, genome-wide STAD miRNA dataset has been made available through the TCGA research network to be used in further research. Our current investigation performed genome-wide screening for identifying underlying prognostic miRNA indicators capable of predicting overall survival (OS) in those patients who are suffering from STAD. We also investigated the potential mechanisms of these prognostic STAD biomarkers.

## Materials and methods

### Data processing

The TCGA STAD level 3 miRNA-sequencing (miRNA-Seq) dataset, as well as relevant clinical parameters were extracted from the TCGA website (https://portal.gdc.cancer.gov/, accessed July 1, 2018) 6. The STAD miRNA raw count dataset was normalized in R by means of *edgeR*. A MiRNAs, which average expression value >1 were fitted into further analysis 21. Patients inclusion criteria includes: (1) those found within the miRNA-Seq dataset; and (2) STAD patients with complete survival information. STAD patients lacking survival information, with a survival time of zero, or not having miRNA expression dataset were excluded from the present study. Owing to the application of open access data extracted from TCGA, this research does not require additional approval by an ethics committee (https://cancergenome.nih.gov/publications/publicationguidelines, accessed July 1, 2018).

### Identification of prognostic related miRNAs

Using the R* survival* package, the prognostic miRNA values were measured through the use of a multivariate Cox proportional hazards regression framework, with adjustments made to the model on the basis of tumor stage. Adjusted* P* value less than 0.05 was recognized as indicating a prognosis-related miRNA of STAD OS.

### MicroRNA expression-based prognostic signature construction

Measured through a “step-wise” function in order to select the optimum screening combination using R, miRNAs were fitted into the prognostic signature screening panel. The combination with the most significant *P* value was considered to be the optimum combination. Furthermore, the weight of prognostic miRNAs in the risk score framework was calculated by multivariate Cox regressive model. The miRNA expression-based prognostic signature, which also named risk score, was created through linear combinations of the expression levels of miRNAs against the weighted multivariable Cox regressive coefficient (β). The formula for determining the risk score was: Risk score = Exp of miRNA_1_×β_1_miRNA_1_+Exp of miRNA_2_×β_2_miRNA_2_+…Exp of miRNA_ n_×β_n_miRNA_n_ (Exp: expression) 22-25. Higher and lower risk patients were determined according to the median value of risk score. The accuracy of risk score model was determined in R through the *survivalROC* package 22, 23.

### Prognosis significance of the risk score model

Stratified survival analysis, as well as joint effects survival analysis was executed in determining the relationship between the clinical features in STAD patients' OS and their associated risk scores. Nomogram was built for assessing the individualized prognostic framework on the basis of clinical parameters and risk score.

### Target gene prediction and assessment of function

Three online tools were applied to determine the target genes of the miRNAs in risk score. These were: TargetScan (http://www.targetscan.org/, accessed July 1,2018) 26, 27, miRDB (http://www.mirdb.org/, accessed July 1,2018) 28, 29, and miRTarBase (http://mirtarbase.mbc.nctu.edu.tw/, accessed July 1,2018) 30, 31. Database tools confirmed the overlying genes targeted by the selected miRNA and were used for downstream analysis. The interaction networks of these miRNA-targeted genes were established through Cytoscape v3.4.0 and the functional assessment was carried out by means of the Database for Annotation, Visualization and Integrated Discovery v6.8 (DAVID v6.8, https://david.ncifcrf.gov/home.jsp, accessed July 1, 2018) 32, 33 and Biological Networks Gene Ontology (BiNGO) tool. Gene-gene interactions and protein-protein interactions were determined by means of GeneMANIA (http://www.genemania.org/, accessed July 1, 2018) and the Search Tool for the Retrieval of Interacting Genes/Proteins (STRING, https://string-db.org/, accessed July 1, 2018), respectively.

### Gene set enrichment analysis (GSEA)

Investigations were performed into the distinctions between functionality of biological process and associated pathways among higher and lower risk miRNA groups in terms of the STAD OS prognostic signature. GSEA (http://software.broadinstitute.org/gsea/index.jsp, accessed July 1, 2018) 34, 35 was used to investigate potential mechanisms in higher and lower risk miRNA groups using the Molecular signs databases (MSigDB) of c2 (c2.all.v6.1.symbols) and c5 (c5.all.v6.1.symbols) 36. Nominal *P*-value less than 0.05 and false discovery rate (FDR) less than 0.25 for each GSEA results were considered statistically significant.

### Statistical analysis

By employing the Benjamini-Hochberg procedure, the FDR in the GSEA was adjusted for multiple testing 37-39. Univariate analysis of the clinical characteristics and OS was performed using the log-rank test. Clinical features with *P* < 0.05 were applied to the multivariate Cox proportional hazards regression model. Moreover, a probability value of *P* less than 0.05 was thought to be statistically significant. SPSS version 20.0 (IBM Corporation, Armonk, NY, USA) and R 3.5.0 (http://www.r-project.org/) was used for performing statistical analysis.

## Results

### Study population and prognosis-related miRNA screening

The STAD patient demographics are summarized in Table 1 and indicate those patients who are more likely to experience an outcome of death due to the diagnosis of advanced tumor stages. There were TCGA data available from 436 patients, and 446 tumor tissue samples and 45 adjacent normal tissue samples. By comparing clinical data to the miRNA-Seq dataset, patients presenting with any of the exclusion criteria were removed from the clinical dataset. In total, 408 patients met study criteria and were included in subsequent survival analysis. A total of 620 miRNAs with a mean value >1, which were used to further investigate their use in prognostic miRNA screening, were derived from the miRNA-Seq dataset. These miRNAs were determined using the *survival* package in R with a multivariate Cox proportional risk regression model with adjustment for tumor stage. The multivariable Cox proportional risk regression model results are shown in Table S1. Following correction for multiple comparison, 41 miRNAs with an adjusted P < 0.05 were found to be associated with prognosis and could be further applied to the construction of a prognostic signature for screening purposes.

### Prognostic signature construction

**Figure 1:**
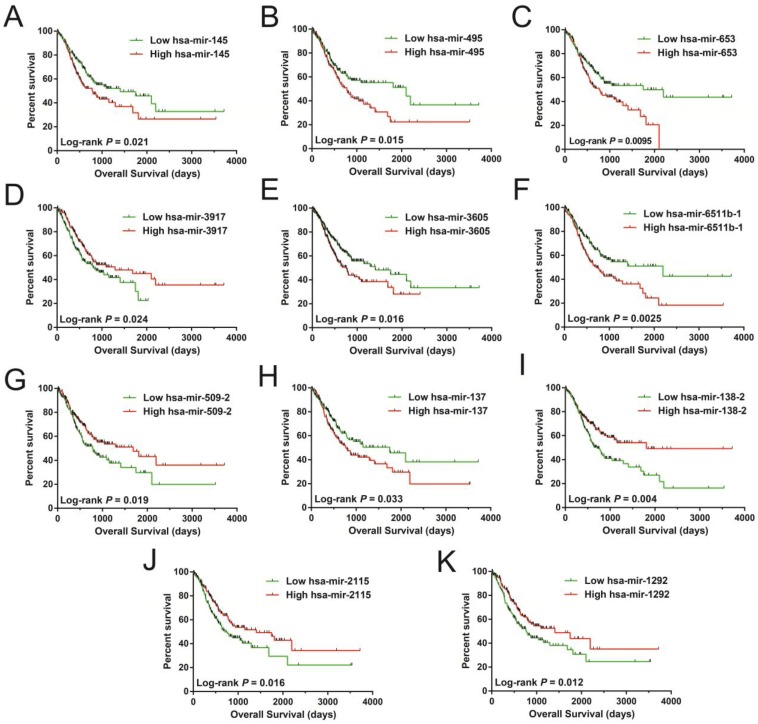
Survival analysis of 11 prognosis-related miRNA sequences in STAD. The sequences are observed as follow: hsa-mir-145 (A), hsa-mir-495 (B), hsa-mir-653 (C), hsa-mir-3917 (D), hsa-mir-3605 (E), hsa-mir-6511b-1 (F), hsa-mir-509-2 (G), hsa-mir-137 (H), hsa-mir-138-2 (I), hsa-mir-2115 (J), and hsa-mir-1292 (K).

**Figure 2:**
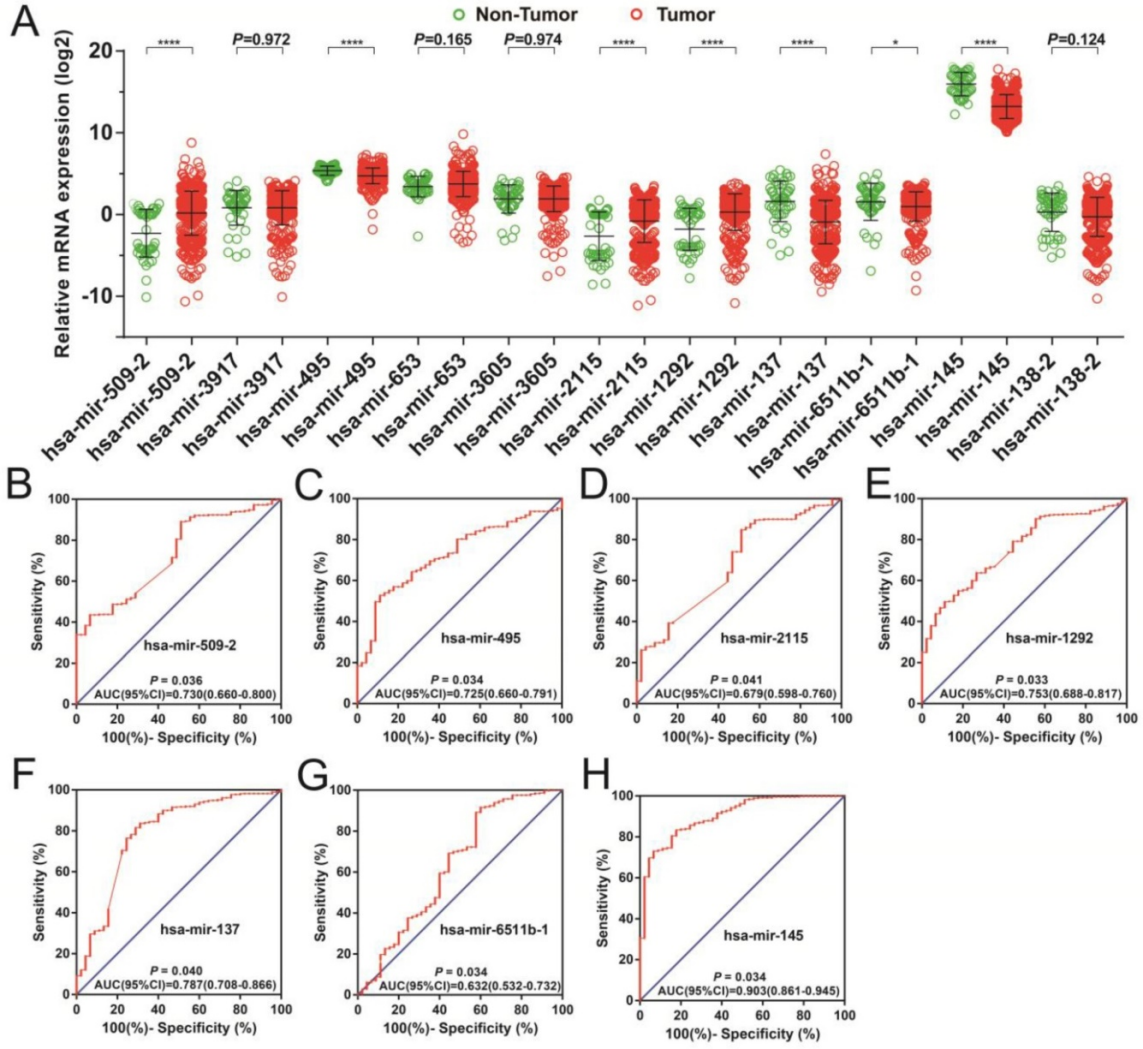
The dispersion of 11 prognostic miRNAs between the tumor and adjacent normal tissue, as well as the investigation into their diagnostic value for STAD. (A) Distribution of expression levels of the 11 prognostic miRNAs between tumor and adjacent normal tissues in STAD. The ROC curves of differentially expressed miRNAs bearing diagnostic value are: hsa-mir-509-2 (B), hsa-mir-495 (C), hsa-mir-2115 (D), hsa-mir-1292 (E), hsa-mir-3917 (F), hsa-mir-6511b-1 (G), and hsa-mir-145 (H).

In order to investigate the optimum combination of the potential prognosis-related miRNAs for screening purposes, the *step* function was performed in R. Each of the associated statistics were log2 transformed for further analysis. Subsequently, the 11 miRNAs that gave rise to most significant *P* values in the prognosis of STAD included: hsa-mir-509-2, hsa-mir-3917, hsa-mir-495, hsa-mir-653, hsa-mir-3605, hsa-mir-2115, hsa-mir-1292, hsa-mir-137, hsa-mir-6511b-1, hsa-mir-145, and hsa-mir-138-2. These miRNA were used for developing a miRNA prognostic model, with the Kaplan-Meier and receiver operating characteristic (ROC) curves shown in Figures 1A-K. In determining the potential diagnostic value of these prognostic miRNAs in distinguishing between STAD tumor and paracancerous tissues, hsa-mir-509-2, hsa-mir-495, hsa-mir-2115, hsa-mir-1292, hsa-mir-137, hsa-mir-6511b-1, and hsa-mir-145 were dysregulated between these tissue types (Figure 2A). ROC analysis demonstrated that hsa-mir-509-2 (Figure 2B), hsa-mir-495 (Figure 2C), hsa-mir-2115 (Figure 2D), hsa-mir-1292 (Figure 2E), hsa-mir-137 (Figure 2F), hsa-mir-6511b-1 (Figure 2G), and hsa-mir-145 (Figure 2H) may serve as potential diagnostic biomarkers for STAD. Multivariate Cox regression analysis was applied for assessing the value of these potential prognostic miRNAs in survival prediction. The formula used for measuring risk is as follows: risk score=Exp of hsa-mir-509-2×(-0.1228)+Exp of hsa-mir-3917×(-0.2394)+Exp of hsa-mir-495×(0.2389)+Exp of hsa-mir-653×(0.1473)+Exp of hsa-mir-3605×(0.2180)+Exp of hsa-mir-2115×(-0.1632)+Exp of hsa-mir-1292×(-0.1974)+Exp of hsa-mir-137×(0.1031)+Exp of hsa-mir-6511b-1×(0.2684)+Exp of hsa-mir-145×(-0.1186)+Exp of hsa-mir-138-2×(-0.1715).

**Figure 3:**
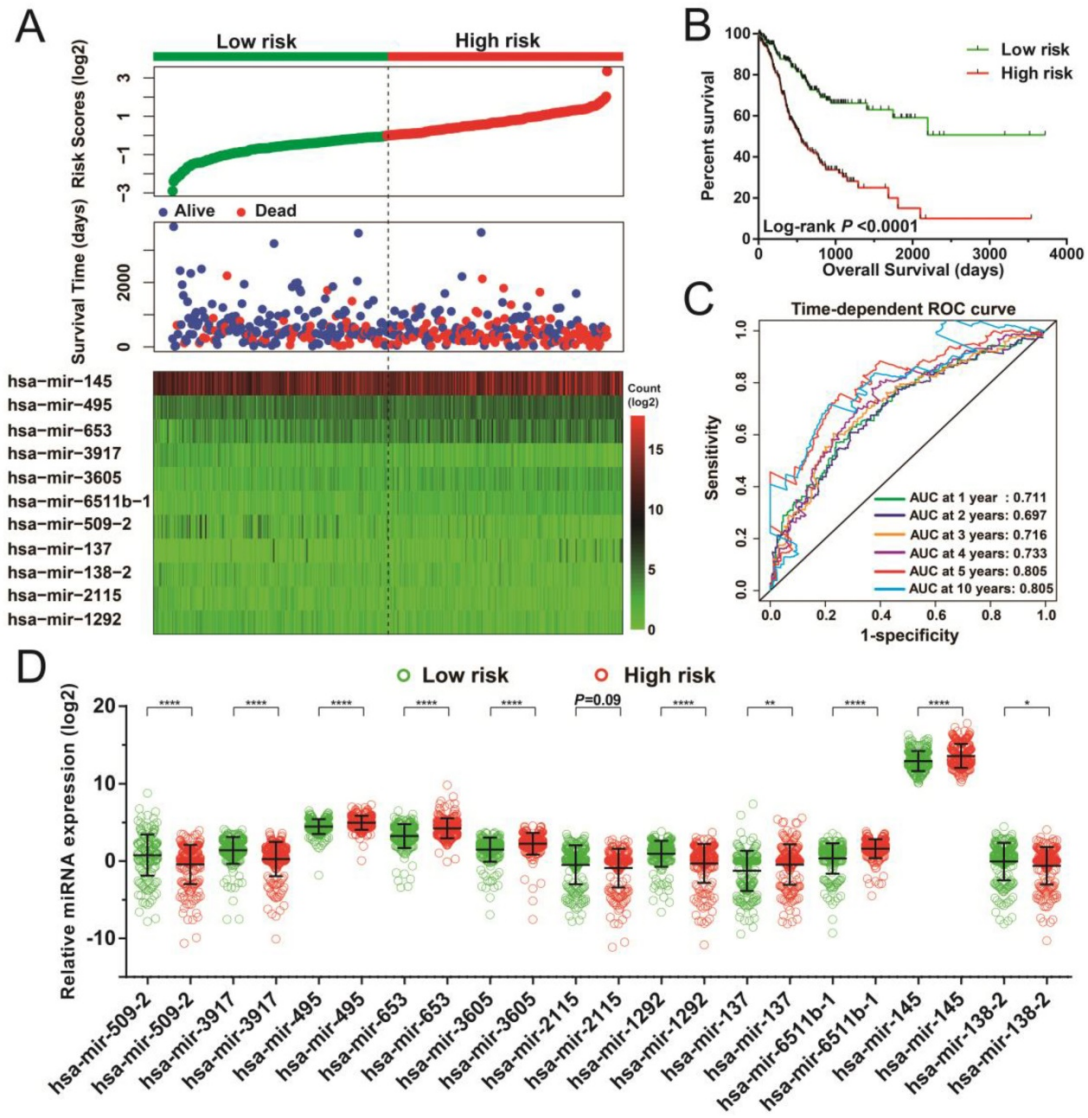
Risk score model of the 11 prognostic miRNAs in STAD patients. (A) Risk score is expressed from top to bottom together with the distribution of the patients' survival status. Heat maps of the 11 prognostic miRNAs expression between the lower- and higher-risk groups are also indicated. (B) Survival analysis between the lower- and higher-risk groups. (C) ROC curve for the determination of the survival rate in patients with STAD based on the risk score. (D) Expression levels of these 11 prognostic miRNAs in groups stratified according to risk score. *P < 0.05, **P < 0.01, ****P < 0.0001.

Following the determination of risk scores and adjustment for tumor stage, those patients who had higher-risk scores showed poorer prognosis, with raised chances leading to death (adjusted *P* < 0.0001, adjusted hazard ratio [HR] = 3.047, 95% confidence interval [CI] = 2.148-4.323 for OS, Figure 3A-B). Moreover, time-dependent ROC examination using the R *survivalROC* package showed that this risk score model could greatly contribute to determining STAD OS. The areas under the curve (AUC) for the time-dependent ROC curves were as follows: 0.711, 0.697, 0.716, 0.733, 0.805, and 0.805 for 1-, 2-, 3-, 4-, 5-, and 10-year survival (Figure 3C), respectively. With the exception of hsa-mir-2115, all remaining miRNAs were differentially expressed between the two risk groups. The miRNA expression profiles between higher and lower risk groups are indicated in Figure 3D.

### Comprehensive survival analysis

**Figure 4:**
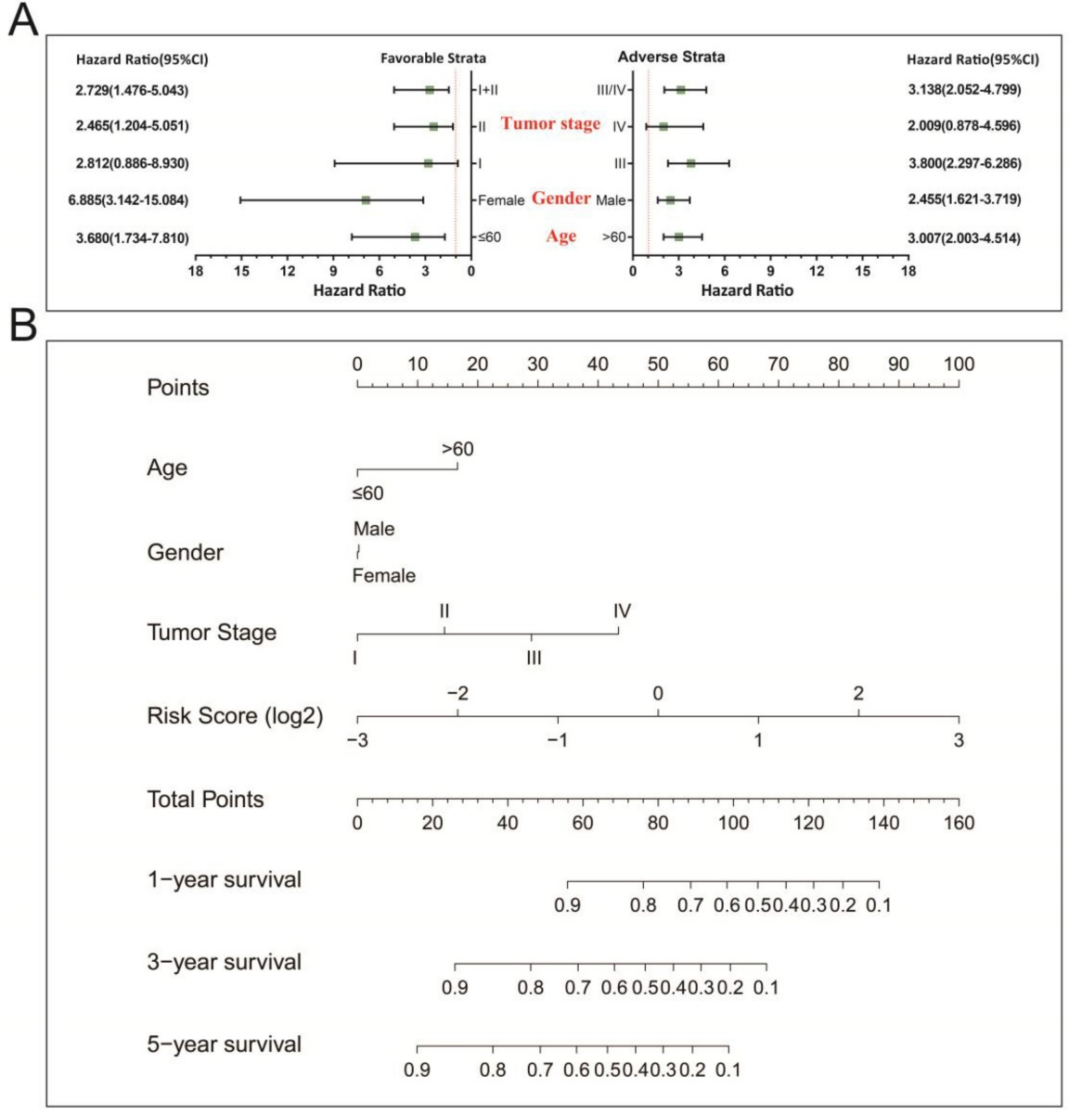
Stratified survival analysis and nomogram for risk score and clinical indicators. (A) Stratified survival analysis for determining the relationship between the risk score and OS in STAD patients. (B) Nomogram of risk scores and clinical indicators for the prediction STAD 1-, 3-, and 5-year events (death).

In a bid to conduct further investigation into the relationship between clinical features and the STAD OS risk score, comprehensive survival examination of nomogram data, together with stratified survival analyses and joint effects survival analyses were performed. Stratified survival analysis opined that a higher risk score remarkably increases the chances of death in those patients who all have favorable strata and all adverse strata. However, this outcome excluded patients who were diagnosed with stage I and IV tumors (Figure 4A). This indicating that this prognostic signature was independent from these clinical features. As determined by a nomogram derived by means of *rms* and its auxiliary datasets, these clinical features contributed to a greater degree in the prognosis prediction of the 11-miRNA signature and the calculated risk points (ranging between 0-100) than other clinical characteristics (Figure 4B).

**Figure 5:**
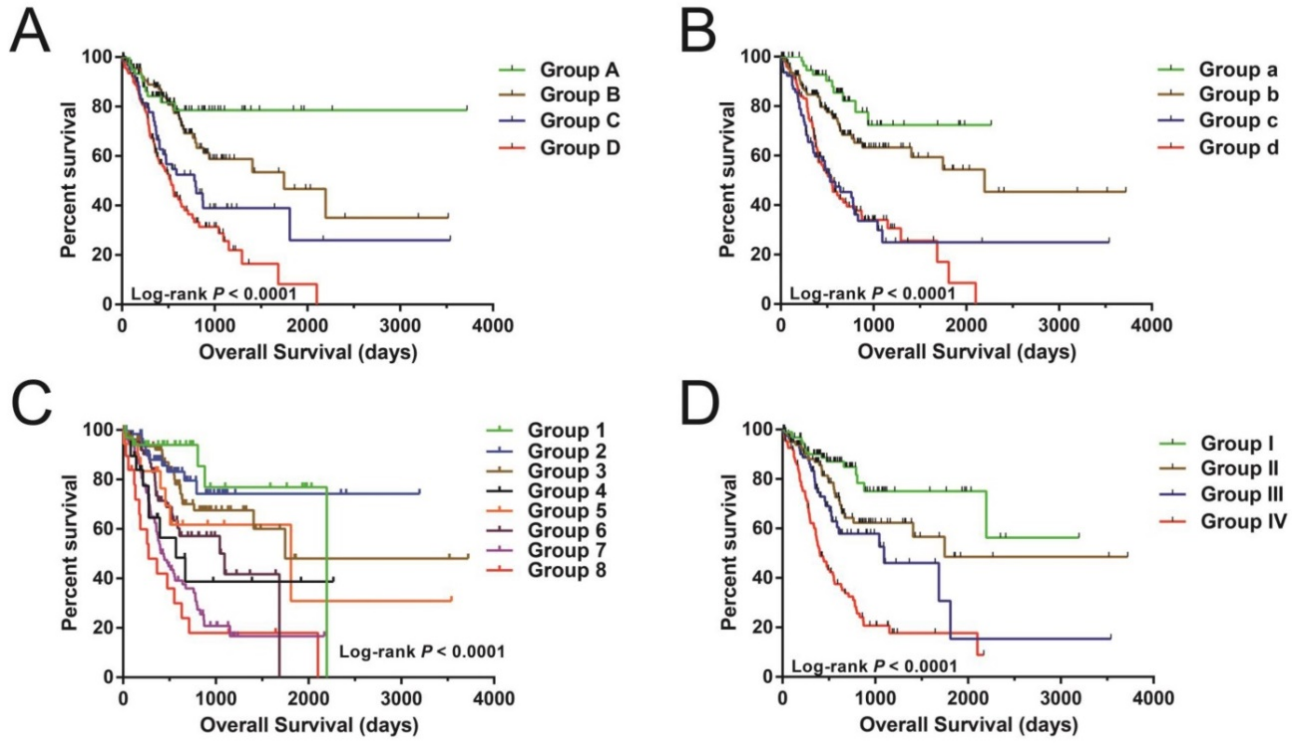
The joint effects analysis of OS when stratifying according to risk score and clinical parameters of STAD. The following parameters were used for joint effects analysis stratification: age (A), gender (B), tumor stage (C), and groupings according to early and advanced tumor stage (D).

Nonetheless, joint effects examination showed that this risk score model performed well in STAD OS prediction and in grouping with conventional clinical indicators (Figure 5A-D and Table 2).

### Target gene prediction and function assessment

**Figure 6:**
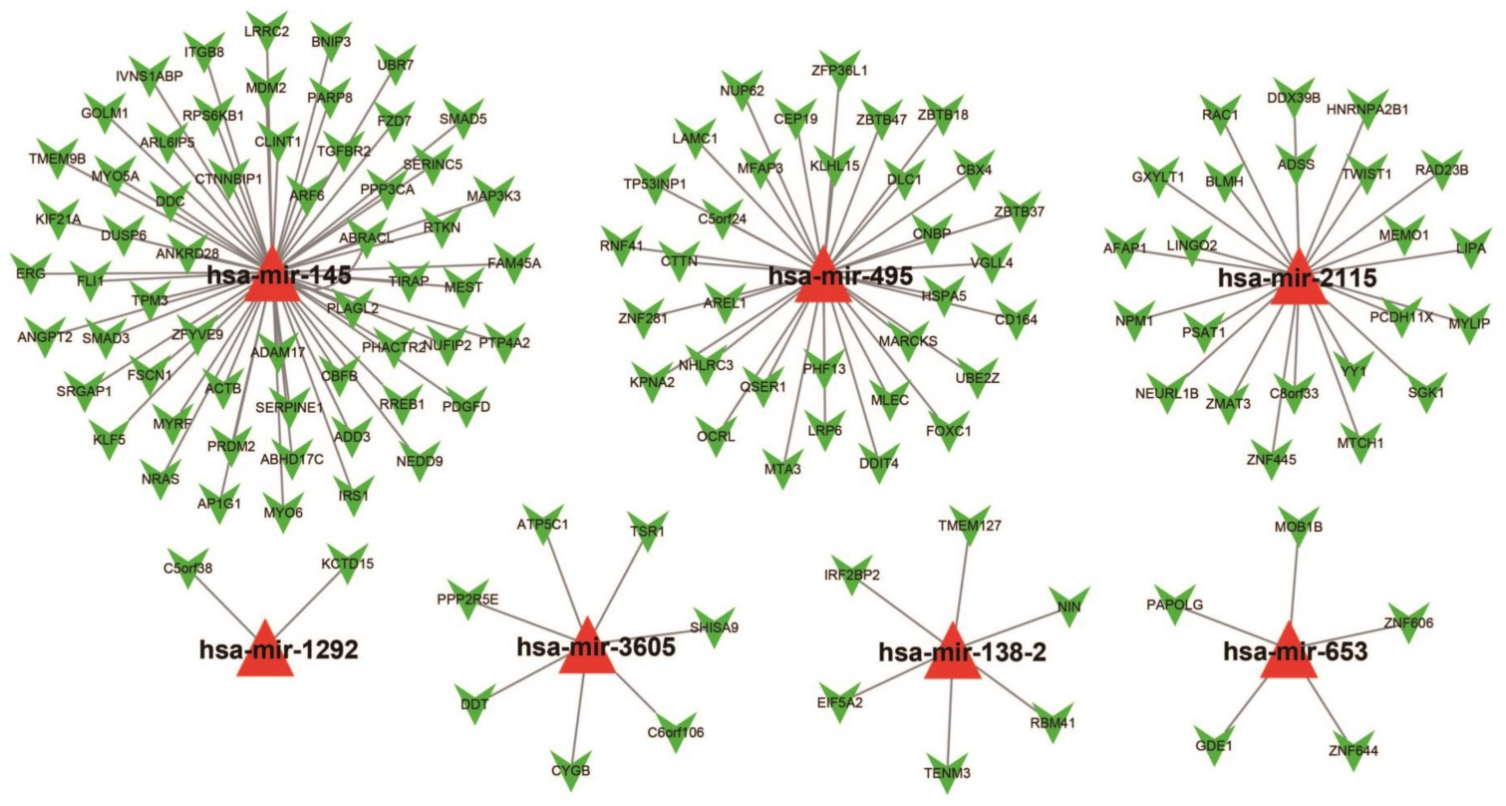
The collaborative network of the 11 miRNAs and their targeted genes. Red triangles indicate the miRNA, green arrows indicate the targeted genes, while the black link shows a miRNA-targeted gene relationship.

For assessing the most probable biological function of these 11 miRNAs, Targetscan, miRDB, and miRTarBase was used. Potential target genes for each miRNA were independently determined in each of these three analysis systems with overlaps in the targeted genes being observed across all three platforms. Target genes associated with only the hsa-mir-495, hsa-mir-653, hsa-mir-3605, hsa-mir-2115, hsa-mir-1292, hsa-mir-145, and hsa-mir-138-2 miRNAs were considered for enrichment examination. There were 134 genes were finally considered as target genes for these seven miRNAs and further applied for the construction of the interactive network (Figure 6).

**Figure 7:**
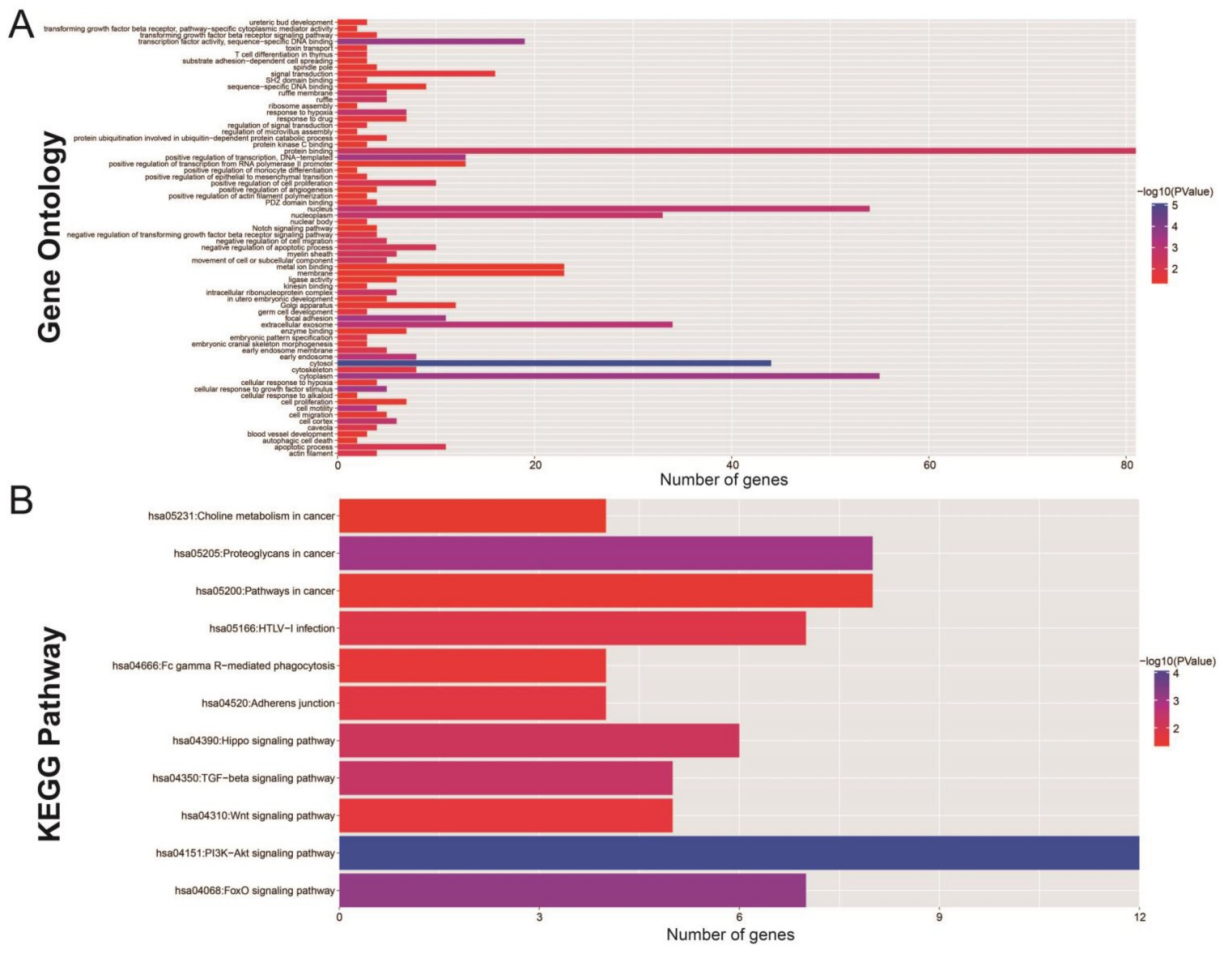
The functional evaluation of the 11 prognostic miRNAs by their targeted genes. (A) GO term analysis of these targeted genes; (B) KEGG analysis of these targeted genes.

The Gene Ontology (GO) analysis indicated that the miRNA-targeted genes are enriched in many biological process, including the regulation of cell proliferation, cell migration, apoptosis and the angiogenesis process, involvement in the Notch signaling pathway, and the transformation of the growth factor beta receptor signaling pathway (Figure 7A). Functional enrichment assessment using BiNGO confirmed these findings (Figure S1).

KEGG (Kyoto Encyclopedia of Genes and Genomes) analysis showed significant enrichment in certain cancer pathways. This included Hippo, transforming growth factor beta (TGFB), Wnt, phosphatidylinositol 3' -kinase (PI3K) -Akt, and the forkhead box O (FoxO) signaling pathways (Figure 7B). Protein-protein (Figure S2) and gene-gene (Figure S3) interactions, performed by STRING and GeneMANIA, additionally demonstrated that these miRNA target genes are involved in complex co-expression interactions.

**Figure 8:**
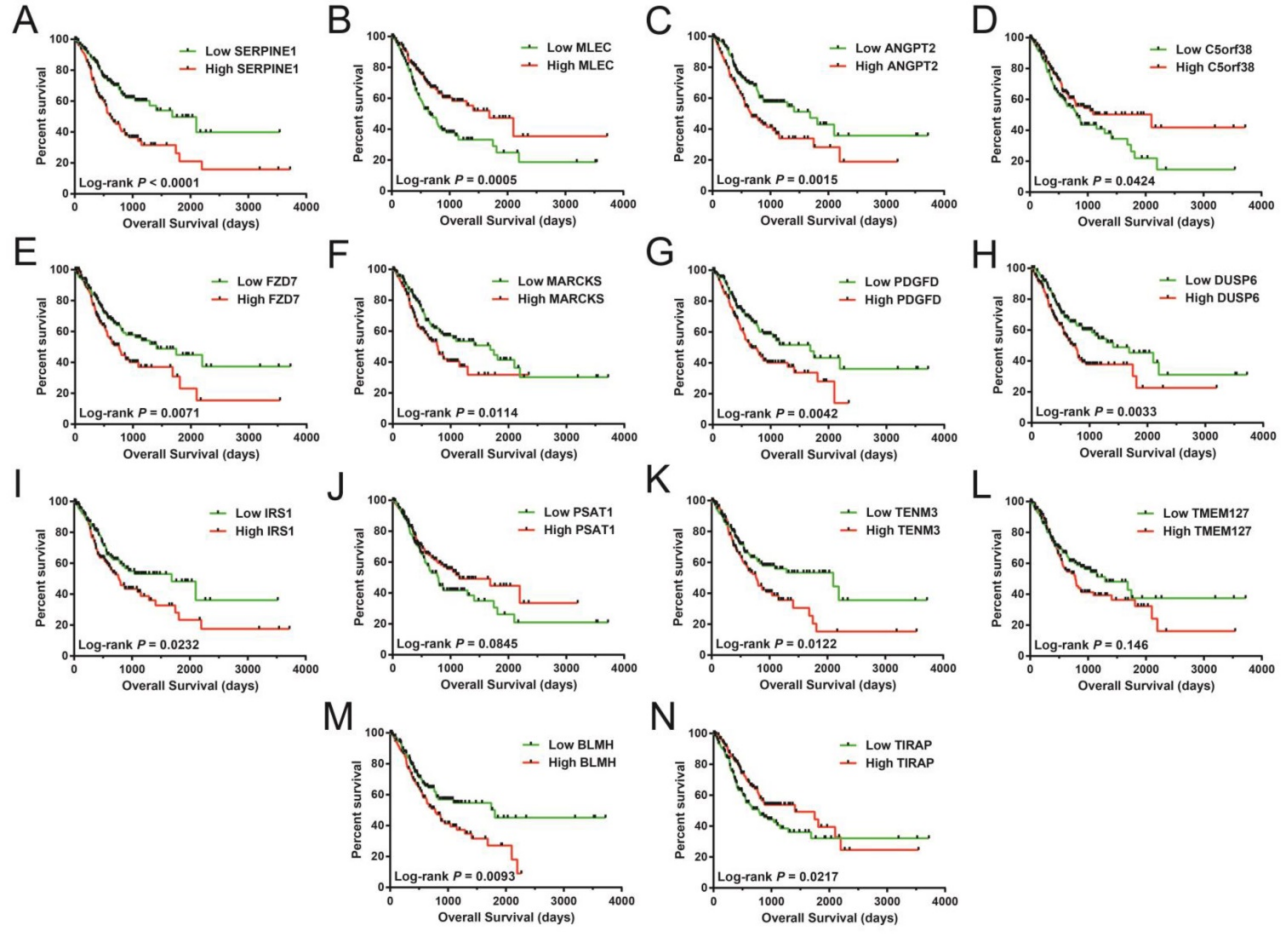
Survival analysis of these targeted genes showed significant association with STAD OS. The order of the Kaplan-Meier curves of these genes is as follows: *SERPINE1* (A),* MLEC* (B), *ANGPT2* (C),* C5orf38* (D),* FZD7* (E), *MARCKS* (F),* PDGFD* (G),* DUSP6* (H),* IRS1* (I), *PSAT1* (J),* TENM3* (K),* TMEM127* (L),* BLMH* (M), and* TIRAP* (N).

In assessing the prognostic value of these miRNA target genes, a multivariable Cox proportionate risk regressive framework was employed. The RNA-sequencing (RNA-Seq) dataset obtained from TCGA website was normalized in R through the *DESeq* package 40. These 14 miRNA-targeted genes were remarkably linked with STAD OS (Table S2), with Kaplan-Meier curves of these genes shown in Figure 8A-N. These 14 prognostic miRNA targeted genes included serpin family E member 1 (*SERPINE1*), malectin (*MLEC*), angiopoietin 2 (*ANGPT2*), chromosome 5 open reading frame 38 (*C5orf38*), frizzled class receptor 7 (*FZD7*), myristoylated alanine rich protein kinase C substrate (*MARCKS*), platelet derived growth factor D (*PDGFD*), dual specificity phosphatase 6 (*DUSP6*), insulin receptor substrate 1 (*IRS1*), phosphoserine aminotransferase 1 (*PSAT1*), teneurin transmembrane protein 3 (*TENM3*), (*TMEM127*), bleomycin hydrolase (*BLMH*), and TIR domain containing adaptor protein (*TIRAP*).

### GSEA

**Figure 9:**
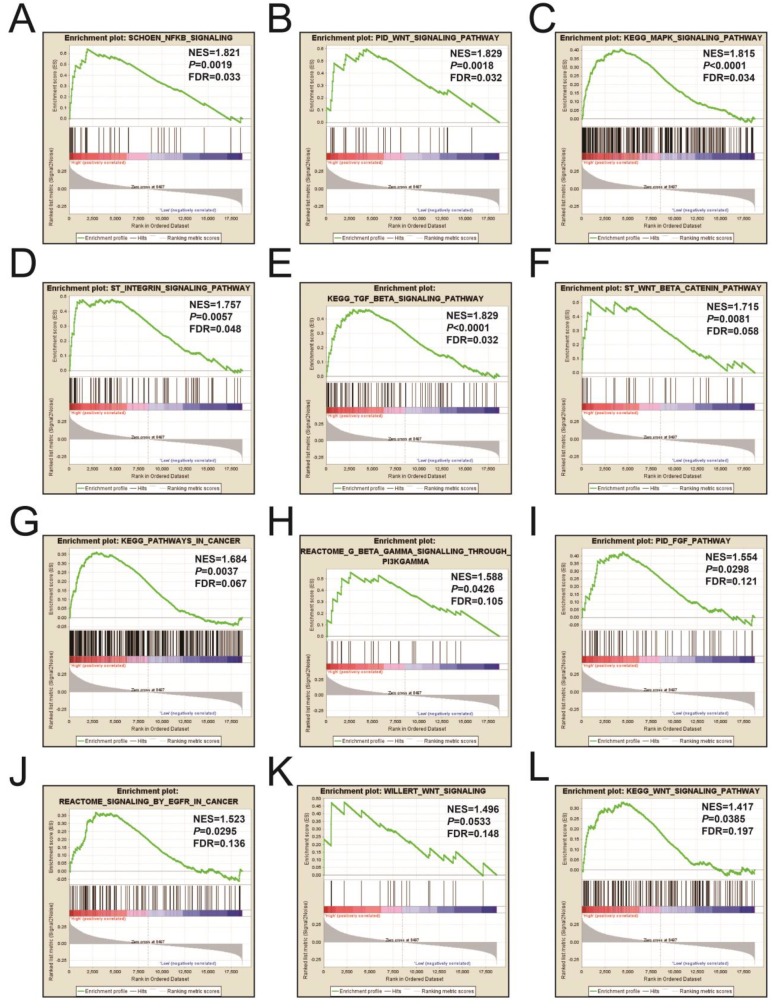
The GSEA findings of the c2 reference gene sets for higher risk groups (A-L).

**Figure 10:**
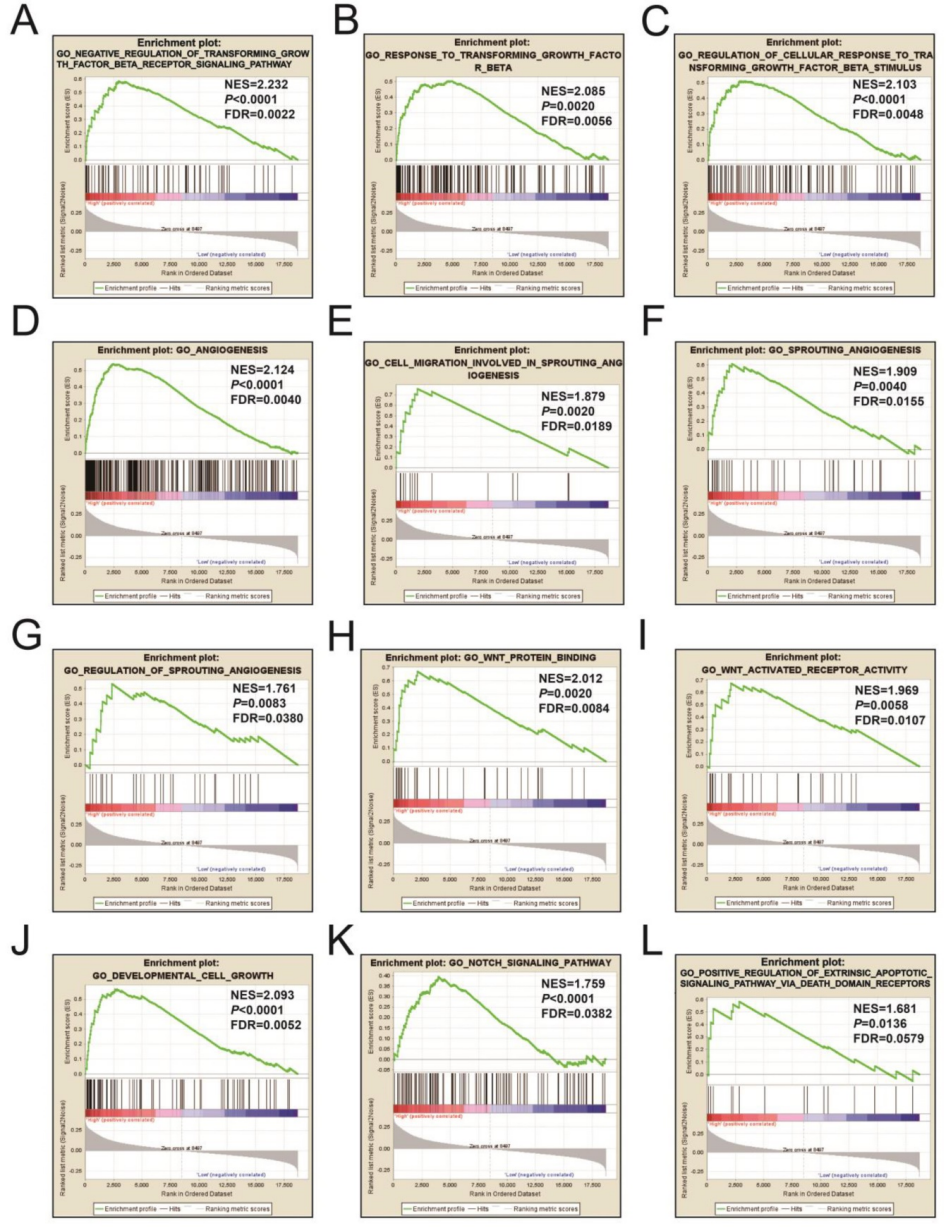
The GSEA findings of the c5 reference gene sets for higher risk groups (A-L).

Figures 9A-L and Table S3 illustrates the GSEA results of the c2 reference gene sets for higher risk-associated groups. Higher risk scores were significantly correlated with Nuclear factor kappa B, Wnt, mitogen-activated protein kinase (MAPK), Integrin, TGFB, PI3K, and fibroblast growth factor (FGF) signaling pathways. These scores were also cancer-associated epidermal growth factor receptor (EGFR) signaling and cancer pathways. **Figures 10A-L** and**Table S4** show the GSEA results of the c5 reference gene sets for higher risk-associated groups. These results indicated that higher risk scores were significantly correlated with the angiogenesis, TGFB, Wnt, Notch, and apoptotic pathways.

## Discussion

It has been stated that miRNA may play a crucial role not only in diagnosing stomach cancer, but also in tumorigenesis, cancer development, and prognostic and targeted biomarker therapies. Previous research has investigated the STAD-associated TCGA miRNA dataset and identified multiple miRNAs as biomarkers for targeted therapy 41, prognosis 15, 42, and disease progression 43. Nevertheless, in their investigations of prognostic biomarkers within the TCGA STAD cohort, these previous studies have only included the miRNAs that were dysregulated among tumor and adjacent normal tissue for further analysis. The advantage of the current study was that a genome-wide screening investigation was performed to identify the potential prognostic miRNAs by using a multivariate Cox proportional risk regression model. Additionally, a risk score model was developed on the basis of miRNA expression, with the potential predictive value in its clinical application for STAD prognosis comprehensively analyzed. Furthermore, the current study used a GSEA approach for the first time not only to explore the differences in biological processes and pathways involved in different patient risk score groups, but also in seeking to clarify their associated molecular mechanisms involved in STAD prognosis. Lastly, 14 potential prognostic miRNA target genes which could serve as biomarkers for STAD OS were identified by employing a multivariate Cox proportional risk regression model.

In this study, 41 miRNAs that were correlated to STAD OS through genome-wide screening were discovered. The sequences of an expression signature comprising hsa-mir-509-2, hsa-mir-3917, hsa-mir-495, hsa-mir-653, hsa-mir-3605, hsa-mir-2115, hsa-mir-1292, hsa-mir-137, hsa-mir-6511b-1, hsa-mir-145, and hsa-mir-138-2, may act as independent biomarkers of STAD OS. Of these 11 miRNAs, the following are related to cancer prognosis or diagnosis: hsa-mir-509-2, hsa-mir-653, hsa-mir-3605, hsa-mir-2115, hsa-mir-1292, and hsa-mir-6511b-1. Moreover, Zhang et al. verified that miR-3917 may act as potential indexes for the early examination of higher-risk inhabitants and earlier diagnosing of lungs cancer 44, while Torres et al. observed that miR-1292 is usually over-expressed in human metastatic colorectal tumor samples and cell lines thereby contributing to a significantly increased risk of death and cancer recurrence 45. Sha et al. summarized the function of miR-138 in tumor biology and concluded that it can act as a tumor suppressor in that its over-expression can inhibit the proliferation of cells, induce apoptosis, inhibit invasiveness and metastasis, and improve chemotherapies that induce drug-persuaded apoptosis 46.

Extensive studies substantiate that miR-495 may function as a tumor suppressor by targeting specific genes, while its overexpression can also inhibit migration activity and invasiveness of tumor-bearing gastric cells 47-49. This tumor suppressive function can also be extended to hepatocellular carcinoma (HCC) 50, colorectal cancer (CRC) 51, non-small cell lung cancer (NSCLC) 52, bladder cancer 53, and prostate cancer 54. However, in this study, we observed that hsa-mir-495 were expressively down-regulated in STAD cancer tissue, and higher expressive levels of hsa-mir-495 were remarkably raised the risk leading to death in those patients who are suffering from STAD. This phenomenon was observed in recent research that contradicts previously published experimental results. Nevertheless, the complexity of the *in vivo* environment and risk factors associated with clinical outcomes may be different from those observed in an *in vitro* environment. Therefore, these results warrant further investigation.

Micro RNA plays a vital role in biological processes, mainly by regulating its target genes. Multiple studies have stated that miR-137 may act as a cancer suppressing agent through the regulation of its target genes, with miR-137 over-expression decreasing the proliferation, invasiveness and metastasis of stomach cancer cells 55-58. However, our current study indicated miR-137 dysregulation between STAD and adjacent normal tissue samples, with STAD tissue being down-regulated, a finding supported by previous gastric cancer studies 55, 59. Additionally, decreased miR-137 expression is correlated to a higher-grade and more advanced tumor phase 55, 60. Gu et al. reported that in 154 gastric cancer patients, lower miR-137 expression levels are significantly correlated to poor clinical outcomes 60. Contrarily, in our study which comprised 408 STAD patients, a higher miR-137 expression level was remarkably associated with poor OS. Since we are unable to provide a reasonable explanation for this contrast, we caution that the prognostic value of miR-137 expression within gastric cancer requires further study.

Various studies have expressed that miR-145 is down-regulated in stomach cancer tissue 43, 61-65. Particularly, the dysregulation of miR-145 can also be found in patients with bladder cancer 66, cervical cancer 67, CRC 68, 69, prostate cancer 70, NSCLC 71, ovarian cancer 72, HCC 73, and endometrioid carcinomas 74. However, usually playing an anti-oncogenic role in gastric cancer, functional experiments indicated that miR-145 over-expression can inhibit the cell cycle, proliferation, migration, invasion, and metastasis of malevolent tumor-bearing gastric cells 63, 65, 75. In determining the value of miR-145 as an indicator for clinical outcome, previous studies reported that higher miR-145 expression levels had remarkably better OS in those patients who are suffering from stomach cancer 43, 61. Conversely, miR-145 with a higher expressed level had remarkably shorter OS, which has been reported in multiple studies of gastric cancer 76-78. For other tumor types, lower miR-145 expression levels had significantly poor prognoses in patients with prostate cancer^79^, ^80^, cervical cancer 67, ovarian cancer^72^, endometrioid carcinomas^74^, and glioblastoma^81^. Our study showed that higher miR-145 expression levels were significantly associated with worse OS in STAD patients. However, due to the limited sample size and an in-depth mechanistic investigation into miR-145-associated gastric cancer, these conflicting results require further exploration.

Furthermore, following miRNA target gene functional enrichment and GSEA analysis, we observed that the expression of the miRNA elements was remarkably enriched for the cell apoptosis, cell growth, angiogenesis, and Wnt and TGFB pathways. These biological processes and pathways can significantly affect the basic state of tumor cells and consequently influence their survival status. Although requiring verification, this may be a potential mechanism associated with the miRNA expression-based signature in STAD prognosis. Anti-angiogenic drugs can be used for targeted therapy of stomach cancer, since angiogenesis related pathway and factors were significantly associated with the clinical outcome of stomach cancer 82. Wnt and its associated downstream pathway play an important role in stomach cancer tumor initiation, growth, metastasis, and resistance to therapy. These biological functions have a great impact on the prognosis of stomach cancer. Wnt pathway antagonists may act as potential target drugs for stomach cancer target therapy 83. Since TGFB pathways can suppress or promote stomach cancer development and progression, they could also be potential therapeutic targets for stomach cancer 84.

This research also has certain limitations that require clarification. Firstly, since all data were taken from a single source, the STAD clinical statistics are not perfect. A lack of information from multiple clinical sources may affect the STAD OS results, consequently giving rise to miRNA-OS relationships that are inconsistent with previous studies. Secondly, since our findings were generated by means of a single cohort and lacked additional cohorts for validation purposes, our results require further verification. Despite these limitations, the advantages of the present study include having conducted a genome-scale miRNA screening from which a prognostic miRNA expression signature associated with STAD OS was identified. This signature may additionally serve as an independent prognosticating predictor for STAD survival. Once the prognostic value of this miRNA expression signature has been validated in a separate, large cohort, it may potentially be clinically applied in STAD surveillance, clinical treatment decision-making and management. Moreover, a larger number of candidate prognostic miRNA markers were identified in the present study and are still worth exploring in the future.

## Conclusions

Our current study performed genome-scale prognostic miRNA screening and identified a large group of candidate prognostic miRNA markers that have the potential to undergo further investigation in STAD patients. More particularly, we determined a novel 11-miRNA expression signature which may serve as a potential prognostic indicator for those patients with STAD. This signature includes hsa-mir-509-2, hsa-mir-3917, hsa-mir-495, hsa-mir-653, hsa-mir-3605, hsa-mir-2115, hsa-mir-1292, hsa-mir-137, hsa-mir-6511b-1, hsa-mir-145, and hsa-mir-138-2. In conducting a comprehensive survival analysis, it was demonstrated that this novel miRNA expression signature was independent from traditional STAD clinical indicators. However, these findings require validation in a large cohort.

## Supplementary Material

Supplementary figure and table legends, figures.

Supplementary tables.

## Figures and Tables

**Table 1:** Correlation between OS and clinical indicators of STAD patients

Variables	Events/total (n=408)	MST (days)	Crude HR (95% CI)	Log-rank *P*
**Age (years)** ₤				0.058
≤60	43/124	1811	1	
>60	120/278	779	1.399(0.986-1.985)	
**Gender**				0.638
Female	55/143	940	1	
Male	109/265	881	1.081(0.781-1.495)	
**Tumor Stage^&^**				<0.0001
Stage I	12/52	2197	1	
Stage II	35/126	1686	1.447(0.750-2.794)	
Stage III	82/174	766	2.530(1.378-4.645)	
Stage IV	24/39	396	4.065(2.031-8.136)	
**Tumor Stage^&^**				<0.0001
Stage I+II	47/178	2197	1	
Stage III+IV	106/213	641	2.130(1.510-3.003)	

**Notes:** ₤ Age information are unavailable in 6 patients; & Tumor stage information are unavailable in 17 patients. OS, overall survival; STAD, colon adenocarcinoma; MST, median survival time; HR, hazard ratio; CI, confidence interval.

**Table 2:** Joint effects survival analysis between clinical indicators and risk score in STAD OS

Group	Risk Score	Variables	Events/total(n=408)	MST (days)	Crude HR (95% CI)	Crude *P*	Adjusted HR (95% CI)	Adjusted *P*£
		Age(years) ₤						
A	Low risk	≤60	11/61	NA	1		1	
B	Low risk	>60	39/138	1747	1.620(0.829-3.166)	0.158	2.207(1.057-4.608)	0.035
C	Low risk	≤60	32/63	782	3.153(1.588-6.258)	0.001	3.623(1.714-7.661)	0.001
D	High risk	>60	81/140	526	4.591(2.435-8.656)	<0.0001	6.296(3.129-12.670)	<0.0001
		**Gender**						
a	Low risk	Female	10/63	NA	1		1	
b	Low risk	Male	40/141	2197	1.910(0.954-3.822)	0.068	2.338(1.087-5.027)	0.030
c	High risk	Female	45/80	533	4.868(2.452-9.666)	<0.0001	6.375(2.981-13.632)	<0.0001
d	High risk	Male	69/124	552	4.790(2.465-9.310)	<0.0001	5.418(2.590-11.334)	<0.0001
		**Tumor Stage^&^**						
1	Low risk	Stage I	5/34	2197	1		1	
2	Low risk	Stage II	11/61	NA	1.433(0.497-4.133)	0.506	1.433(0.497-4.133)	0.506
3	Low risk	Stage III	21/83	1747	1.958(0.738-5.199)	0.177	1.958(0.738-5.199)	0.177
4	Low risk	Stage IV	9/20	570	4.547(1.522-13.584)	0.007	4.547(1.522-13.584)	0.007
5	High risk	Stage I	7/18	1811	3.145(0.997-9.920)	0.051	3.145(0.997-9.920)	0.051
6	High risk	Stage II	24/65	1043	3.642(1.384-9.586)	0.009	3.642(1.384-9.586)	0.009
7	High risk	Stage III	61/91	439	7.236(2.895-18.087)	<0.0001	7.236(2.895-18.087)	<0.0001
8	High risk	Stage IV	15/19	274	9.660(3.505-26.622)	<0.0001	9.660(3.505-26.622)	<0.0001
		**Tumor Stage^&^**						
I	Low risk	Stage I+II	16/95	NA	1		1	
II	Low risk	Stage III+IV	30/103	1747	1.873(1.020-3.4360	0.043	3.154(1.380-7.212)	0.006
III	High risk	Stage I+II	31/83	1095	2.793(1.525-5.116)	0.001	2.701(1.466-4.974)	0.001
IV	High risk	Stage III+IV	76/110	401	6.049(3.517-10.406)	<0.0001	10.172(4.657-22.216)	<0.0001

**Notes**: £Adjusted for tumor stage. ₤ Age information are unavailable in 6 patients; & Tumor stage information are unavailable in 17 patients. OS, overall survival; STAD, colon adenocarcinoma; MST, median survival time; HR, hazard ratio; CI, confidence interval.

## Abbreviations

STAD: stomach adenocarcinoma
TCGA: The Cancer Genome Atlas
OS: overall survival
miRNA-Seq: miRNA-sequencing
DAVID: Database for Annotation, Visualization and Integrated Discovery
BiNGO: Biological Networks Gene Ontology tool
STRING: Search Tool for the Retrieval of Interacting Genes/Proteins
GSEA: Gene set enrichment analysis
FDR: false discovery rate
AUC: area under curve
HR: hazard ratio
CI: confidence interval
GO: Gene Ontology
KEGG: Kyoto Encyclopedia of Genes and Genomes
