# Supplementary material for: Genome-wide analysis to identify a novel microRNA signature that predicts survival in patients with stomach adenocarcinoma
Source: J Cancer. 2019 Oct 17;10(25):6298–313. doi: 10.7150/jca.33250 (PMC6856753; doi:10.7150/jca.33250)
Supplement: Supplementary file 1 — Supplementary figure and table legends, figures. [file jcav10p6298s1.pdf]

Supplementary material

Supplementary Tables

Table S1. Illustrated the multivariable survival analysis results of miRNAs.

Table S2. Illustrated the multivariable survival analysis results of these prognostic miRNAs targeted genes.

Table S3. Showed the GSEA results of c2 (c2.all.v6.1. symbols) reference gene sets for higher risk groups in those patients who are suffering from STAD.

Table S4. Showed the GSEA results of c5 (c5.all.v6.1.symbols) reference gene sets for higher risk groups in those patients suffering from STAD.

Supplementary Figures

Figure S1. Directed acyclic graph of miRNA-targeted gene that generated by BiNGO.

Figure S2. Protein–protein interaction networks of miRNA-targeted gene that generated by SRTING.

Figure S3. Gene-gene interaction networks of miRNA-targeted gene that generated by GeneMANIA.

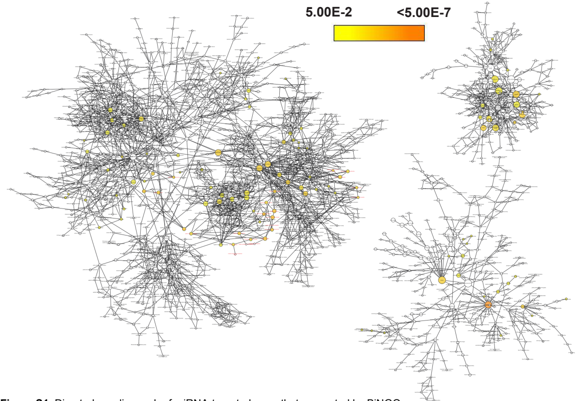

Figure S1. Directed acyclic graph of miRNA-targeted gene that generated by BiNGO.

Figure S2. Protein–protein interaction networks of miRNA-targeted gene that generated by SRTING.

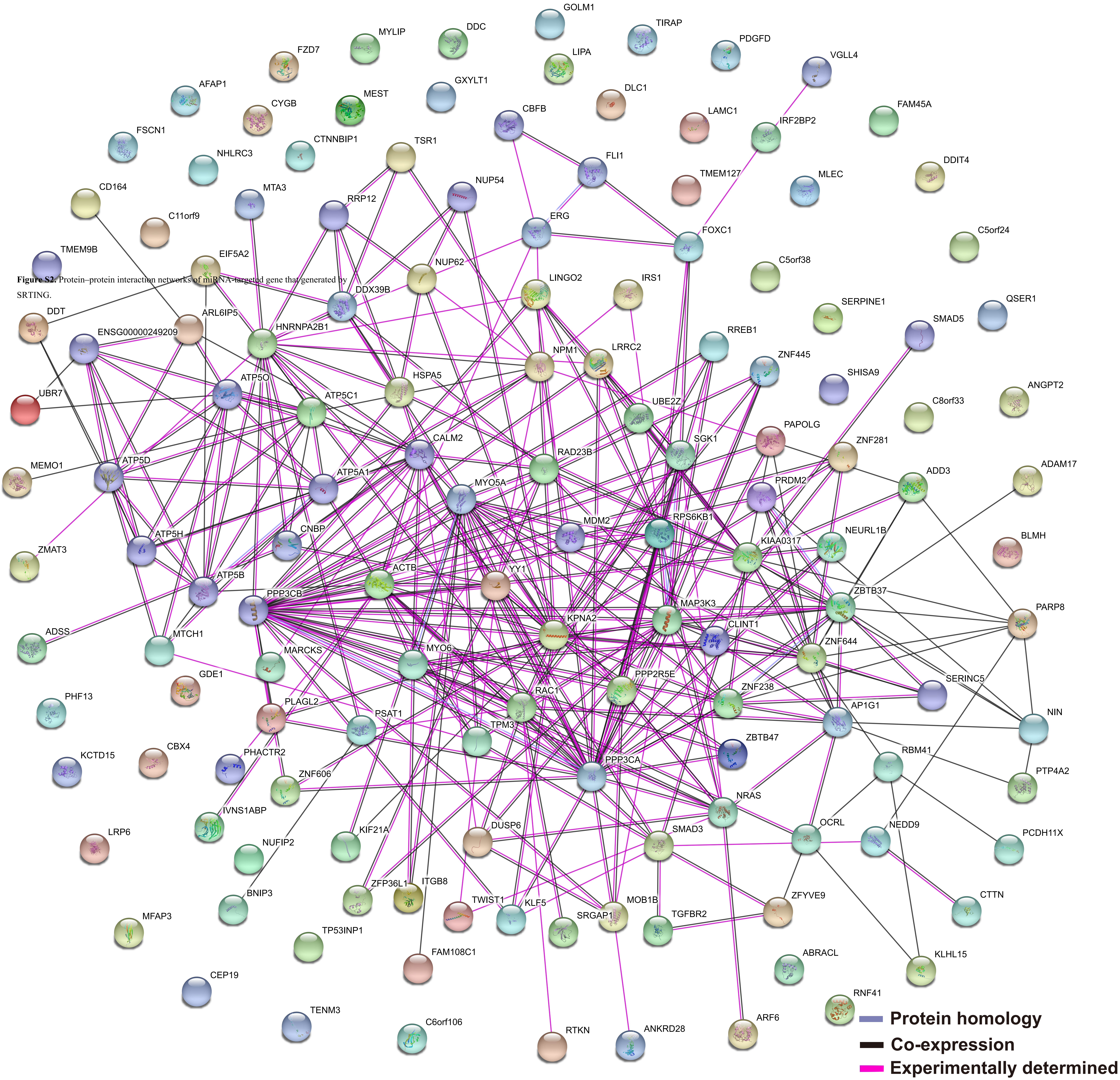

Figure S2. Protein–protein interaction networks of miRNA-targeted gene that generated by SRTING.

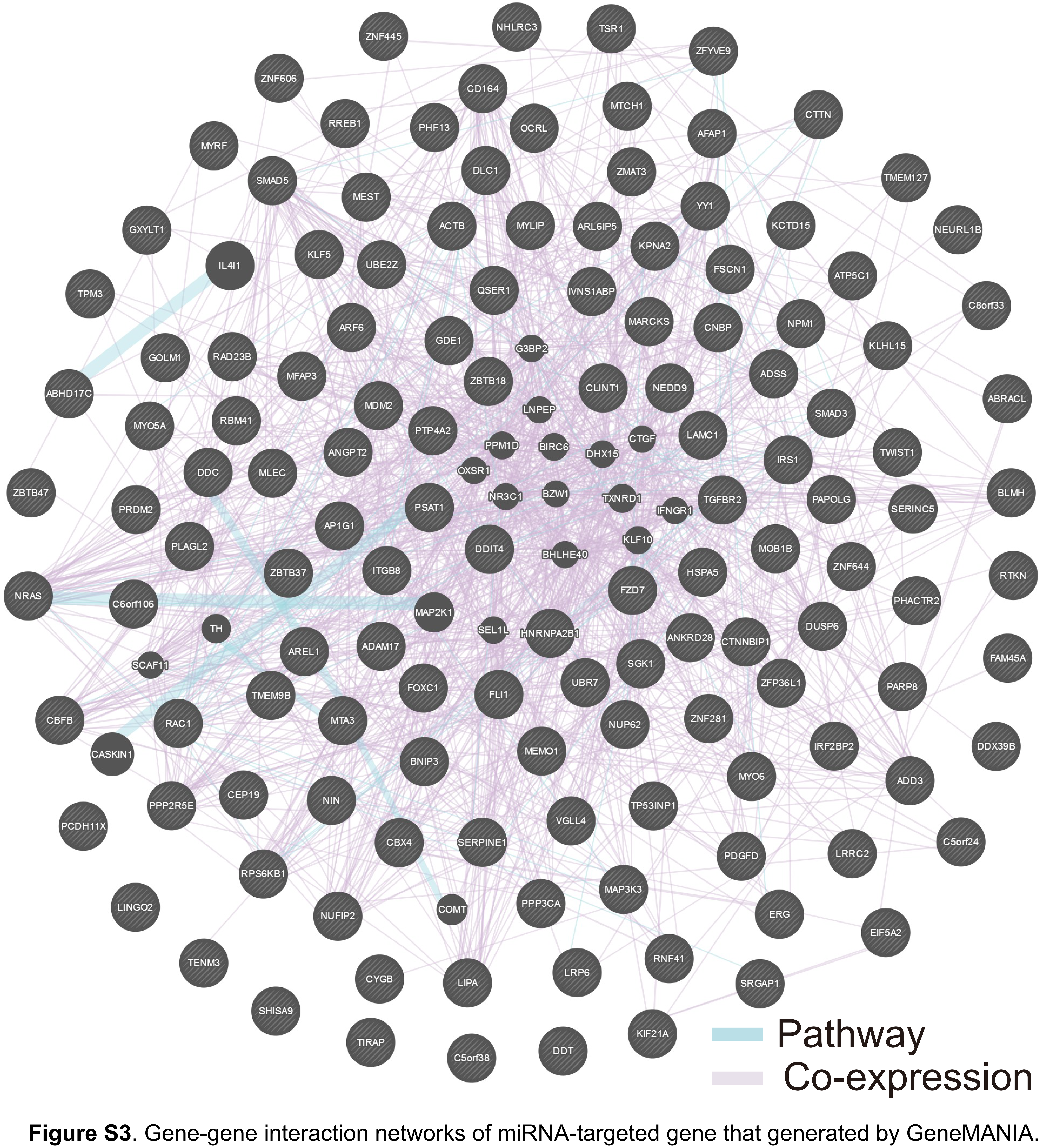

Figure S3. Gene-gene interaction networks of miRNA-targeted gene that generated by GeneMANIA.
